# 1585. Efficacy and safety of long-acting subcutaneous lenacapavir in heavily treatment-experienced people with multi-drug resistant HIV: Week 52 results

**DOI:** 10.1093/ofid/ofac492.108

**Published:** 2022-12-15

**Authors:** Onyema Ogbuagu, Sorana Segal-Maurer, Winai Ratanasuwan, Benoit Trottier, Jason Brunetta, Takuma Shirasaka, Ellen L Koenig, Hui Wang, Nicolas A Margot, Hadas Dvory-Sobol, Martin S Rhee, Jared Baeten, Jean-Michel Molina

**Affiliations:** Yale School of Medicine, New Haven, Connecticut; Division of Infectious Diseases, New York–Presbyterian Queens, Flushing, New York; Faculty of Medicine, Siriraj Hospital, Mahidol University, Bangkok, Krung Thep, Thailand; Clinique de medecine urbaine du Quartier Latin, Montreal, Quebec, Canada; Maple Leaf Research, Toronto, Ontario, Canada; National Hospital Organization Osaka National Hospital, Osaka, Osaka, Japan; Instituto Dominicano de Estudios Virologicos (IDEV), Santo Domingo, Distrito Nacional, Dominican Republic; Gilead Sciences Inc., Foster City, California; Gilead Sciences Inc., Foster City, California; Gilead Sciences, Foster City, California; Gilead Sciences, Foster City, California; Gilead Sciences, Foster City, California; UNiversity of Paris CIte, Paris, Ile-de-France, France

## Abstract

**Background:**

Lenacapavir (LEN), a potent first-in-class inhibitor of HIV-1 capsid function, is in development as a long-acting agent for treatment and prevention of HIV-1.

**Methods:**

CAPELLA is an ongoing, phase 2/3 study in heavily treatment-experienced (HTE) people with HIV-1 (PWH) with multidrug-resistance. 36 participants were randomized (2:1) to add oral LEN or placebo to their failing regimen. At D15, those on oral LEN received subcutaneous (SC) LEN 927 mg (Q6M); those on placebo started the oral LEN lead-in, followed by SC Q6M. All randomized participants initiated an investigator-selected, optimized background regimen (OBR) at D15. An additional 36 participants started OBR concurrent with LEN (oral lead-in → SC) in a non-randomized cohort. We report the Week (W) 52 efficacy and safety results from both cohorts.

**Results:**

Of 72 participants enrolled, 25% were female, 38% Black, median age 52 years, 64% had CD4 < 200 cells/µL, 46% had HIV-1 resistant to all 4 major classes (NRTI, NNRTI, PI, INSTI), and 53% had OBR with 1 or no fully active agents. At W52, 78% (56/72) achieved VL< 50 c/mL and 82% (59/72) achieved VL< 200 c/mL via FDA Snapshot algorithm. CD4 count increased by a median 84 cells/µL (Q1 to Q3: 21 to 153) and the proportion of participants with CD4 count ≥200 cells/ul increased from 36% at baseline to 68% at W52. Ten participants had emergent LEN resistance (8 previously reported); 4 of 10 subsequently suppressed. The median (range) duration of follow up on LEN was 71 (13–111) weeks. One participant discontinued due to injection site nodule (Grade 1). The most common injection site reaction (ISR) was swelling (28% [20/72] and 17% [12/70] after the 1^st^ and 2^nd^ SC doses, respectively). Most ISRs were mild or moderate. The most common AEs (excluding injection site reactions) were nausea and diarrhea (14% each).

**Conclusion:**

In HTE PWH, subcutaneous LEN was well tolerated and in combination with OBR led to high and sustained rate of virologic suppression at W52. These results support the potential role for LEN for treatment of multi-drug resistant HIV-1 infection.

**Disclosures:**

**Sorana Segal-Maurer, MD**, Gilead Sciences: Advisor/Consultant|Gilead Sciences: Grant/Research Support|Gilead Sciences: Honoraria|JANSSEN THERAPEUTICS: Honoraria|ViiV: Honoraria **Benoit Trottier, MD**, Gilead Sciences: Advisor/Consultant|Gilead Sciences: Honoraria|Merck: Advisor/Consultant|Merck: Honoraria|ViiV Healthcare: Advisor/Consultant|ViiV Healthcare: Honoraria **Jason Brunetta, M.D.**, Gilead Canada: Advisor/Consultant|Gilead Canada: Honoraria|Gilead Canada: Conference Attendance Sponsorship|Viiv Canada: Advisor/Consultant **Hui Wang, PhD**, Gilead Sciences: Employment|Gilead Sciences: Stocks/Bonds **Nicolas A. Margot, MA**, Gilead Sciences: Employment|Gilead Sciences: Stocks/Bonds **Hadas Dvory-Sobol, PhD**, Gilead Sciences: Employment|Gilead Sciences: Stocks/Bonds **Martin S Rhee, MD**, Gilead Sciences: Stocks/Bonds **Jared Baeten, MD, PhD**, Gilead Sciences: Employee|Gilead Sciences: Stocks/Bonds **Jean-Michel Molina, MD; PhD**, GIlead: Board Member|GIlead: Grant/Research Support|Merck: Board Member|Merck: Expert Testimony|ViiV: Board Member.

